# Defining and assessing vulnerability within law enforcement and public health organisations: a scoping review

**DOI:** 10.1186/s40352-019-0083-z

**Published:** 2019-03-01

**Authors:** Iniobong Enang, Jennifer Murray, Nadine Dougall, Andrew Wooff, Inga Heyman, Elizabeth Aston

**Affiliations:** 1 School of Health & Social Care, Edinburgh Napier University, Sighthill Campus, Sighthill Court, Edinburgh, EH11 4BN Scotland, UK; 2 School of Applied Sciences, Edinburgh Napier University, Sighthill Campus, Sighthill Court, Edinburgh, EH11 4BN Scotland, UK

**Keywords:** Law enforcement, Public health, Policing, Vulnerability, Vulnerability assessment

## Abstract

**Background:**

Historically, police departments focused solely on criminal justice issues. Recently, there has been a dynamic shift in focus, with Law Enforcement professional groups assuming more responsibility for tackling mental health and distress-related issues (that may arise because of mental health related problems and learning disabilities) alongside Public Health departments. While Law Enforcement has become a ‘last line of support’ and an increasing partner in mental health support, there is partnership working between law enforcement, psychology, and health professions in training and mental health service delivery. The term vulnerability is frequently used across Law Enforcement and Public Health (LEPH) to identify those in need of these services. Effective vulnerability assessment is therefore expected to prevent unintentional harmful health and criminal justice consequences and manage the negative impact of such in cases where prevention is not possible. This scoping review aimed to identify how vulnerability is defined and assessed across LEPH organisations.

**Results:**

Vulnerability is context-specific from a Law Enforcement perspective, and person-specific from a Public Health perspective. Definitions of vulnerability are at best fragmented, while models for assessing vulnerability lack uniformity across LEPH. The implications are two-fold. For “vulnerable groups”, the lack of an evidence-based definition and assessment model could prevent access to relevant LEPH services, exacerbating issues of multiple vulnerabilities, co-morbidity, and/or dual diagnosis. All could inadvertently enable social exclusion of vulnerable groups from political discourse and policy interventions. The lack of consistency regarding vulnerability may result in reactive crisis responses as opposed to proactive preventative measures.

**Conclusions:**

This scoping review exposes the complexities associated with defining and assessing vulnerability from a LEPH perspective, which are perceived and prioritised differently across the organizations. Future research must bridge this gap. Building on the establishment of a definition of vulnerability within the empirical literature, researchers ought to engage with service users, LEPH staff, and those engaged in policy making to craft effective vulnerability definitions and assessment models. Only through evidence based, co-produced definitions and assessment models for vulnerability can we ensure that best-practice, but also meaningful and feasible practice, in vulnerability assessment can be achieved.

## Background

There is increasing international recognition that greater emphasis on partnership working across the intersect of policing and public health is a necessity (Police Scotland, 2017; Punch & James, 2017). Despite the different contexts in which policing (traditionally linked to criminal justice and establishing law and order) and public health (provision of physical, mental, and social well-being) operate, both fields share similar complex challenges; necessitating closer partnership working between them (Van Dijk & Crofts, 2017). One of these complex challenges relates to vulnerability.

Vulnerability has been defined in different ways, depending upon the field and literature being discussed. One example which offers an ‘all encompassing’ perspective outlines vulnerability as a state or condition whereby a person is in danger, under threat, experiencing health challenges, at risk, and/or requiring support/protection (Larkin, 2009). This definition suggests, then, that anyone can be vulnerable at any point in time, and that vulnerability is not a stable state across situations and the lifespan. While this is a useful way to consider vulnerability – as a holistic, variable construct – it may be considered too broad a construct to then develop assessment strategies and protocols, form policies, and indeed understand within the specific remit of law enforcement and public health.

Vulnerability is a key concern across policing and public health partners (Murray et al., 2018), with increasing prioritisation being given to the identification, assessment, and management of vulnerable victims and perpetrators of crime (College of Policing, 2018; Department of Health, 2014). Indeed, Police Scotland Strategy 2026 notes that top priority is to protect vulnerable people (Police Scotland, 2017). Despite this, there appears to be no unified definition of vulnerability across policing and public health practices, or within the policy documentation or literature. This, then, inhibits our understandings of what vulnerability means at the intersect of policing and public health, and makes the identification, assessment, and management of vulnerable people challenging for police and health professionals. Equally, should a unified understanding and shared definition of vulnerability be established and adopted across the intersect of policing and public health, communication, decision making, and management of vulnerable people with complex needs across the criminal justice and health systems could be improved.

The current scoping review aims to identify how vulnerability is defined and assessed in relation to the adult population across Law Enforcement and Public Health (LEPH). It focuses on collaborative partnership working across LEPH. For the purposes of the current review, we will use ‘Law Enforcement’ in a broad sense, recognising that the role of law and policing professionals is much broader than enforcement. We therefore adopt the broader context of the role, including working with the public and other partners, community engagement, etc. Public Health, again, adopts a broad definition, including any health and social care professional who works with individuals who could be considered or who consider themselves as vulnerable.

Scoping reviews are conducted for a variety of reasons including: conceptual mapping^1^ (Anderson et al., 2008); literature mapping^2^ (Anderson et al., 2008; Ehrich et al., 2002); policy mapping^3^(Anderson et al., 2008); and identification of research gaps (Arksey & O’Mally, 2005), including the extent and nature of research evidence (Grant et al., 2009). From a LEPH perspective, the current review was required and carried out to address three interrelated issues. First, to conceptually map and lend understanding to how the term ‘vulnerability’ is defined and the context in which it is used in different countries and LEPH organizations (Anderson et al., 2008). Second, to identify the models or methods of vulnerability assessment as presented in these documents (Grant et al., 2009). Building on the first and second aims, the third seeks to identify under-researched areas within the context of vulnerability assessment in LEPH (Ehrich et al., 2002) to identify key research priorities for future research in vulnerability and assessment across LEPH.

The selected methodological approach aligns with Arksey and O’Mally’s (2005) six stage framework, and incorporates recommendations provided by Levac et al. (2010). The stages of the framework are: identifying a research question; finding appropriate studies; selecting the studies; conducting content analysis via the synthesis and interpretation of qualitative data; organizing, summarizing and recording results; and stakeholder consultation. Discussions within the current review are structured according to these sections for the readers’ ease.

## Methods

### Stage 1: Identifying a research question

The central research question for the current review asks: “What can we learn from extant literature about how LEPH professional groups define and assess vulnerability within the adult population?”

The central research question was divided into two sub-questions to ensure that the critical elements (vulnerability definition and vulnerability assessment) of the study were effectively addressed:

1. From a LEPH perspective how is vulnerability defined within the adult population?
2. Considering this demographic, do models for vulnerability assessment exist within or across LEPH professional groups?

### Stage 2: Finding appropriate studies

**Fig. 1:**
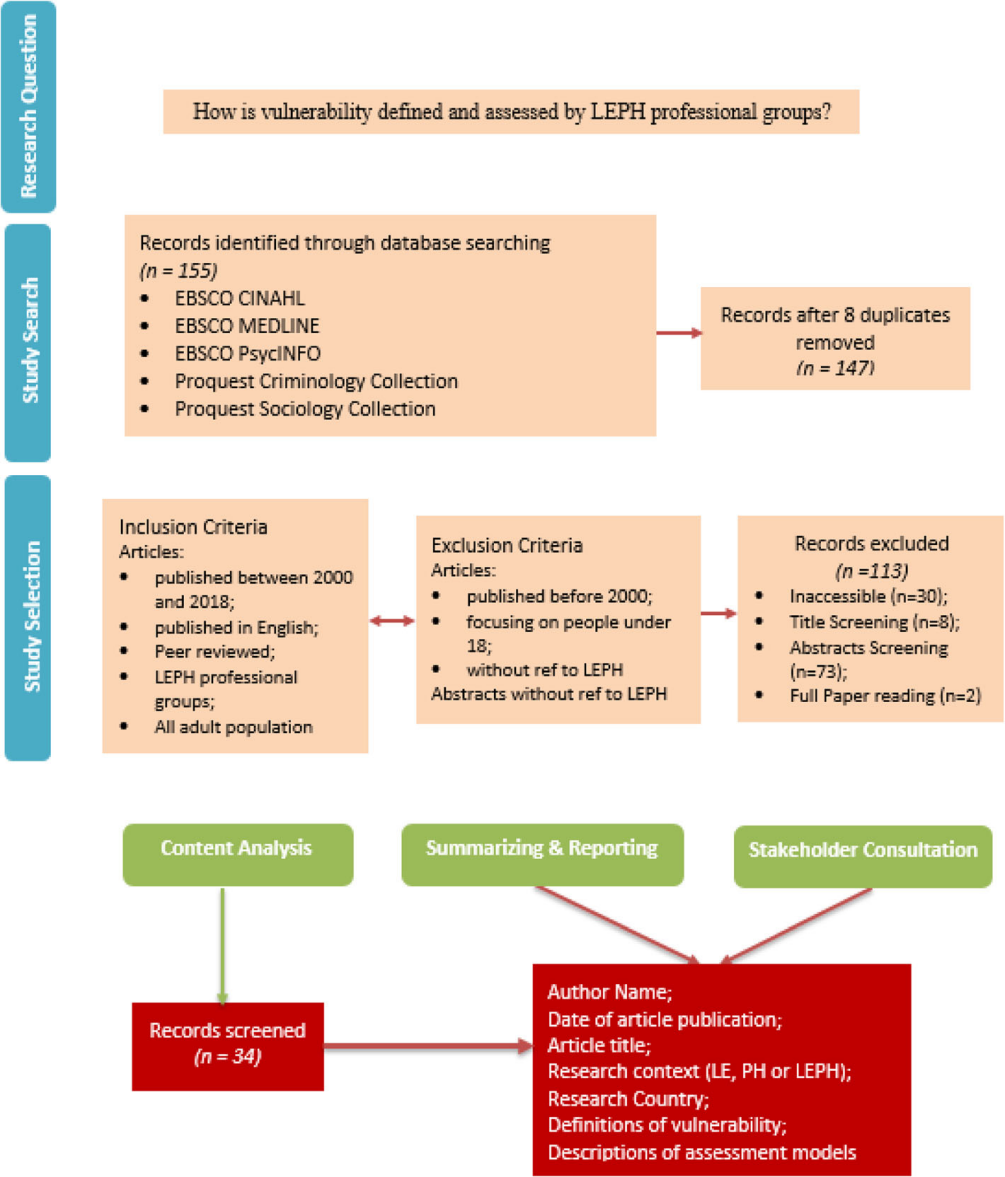
Overview of scoping review process

Following the identification of the research question and sub-questions, the next step entailed finding appropriate studies. To this end, inclusion and exclusion criteria were developed as presented in Fig. 1 and Table 1.

**Table 1:** Inclusion and Exclusion Criteria

Inclusion Criteria	Exclusion criteria
Articles published in English	Articles published in a language other than English
Articles published between 2000 and 2018	Articles published before 2000
Articles discussing vulnerability and vulnerability assessment	Abstracts without reference to vulnerability
All adult population (> 18 y/o)	Children and young people below 18 years old
LEPH professional groups in any country	Articles without references to LEPH professional groups
Articles retrieved from five key databases: Cumulative Index of Nursing and Allied Health Literature (CINAHL), Medical Literature Analysis and Retrieval System Online (MEDLINE), Psychological Information Database (PsycINFO), Criminology Collection, and Sociology Collection	Book chapters and non-peer reviewed articles

#### Inclusion criteria

As indicated in Table 1, articles included for review were published in English, between the years 2000–2018. The year 2000 was selected because the Adults with Incapacity (Scotland) Act 2000 was passed then (The Scottish Government, 2008) and the research team are based within Scotland hence its contextual relevance.

The date of publication of this legislation in Scotland was key as it led the way towards recognising the limitations faced by adults with mental health challenges across LEPH contexts. It is instrumental to the current review because mental health problems are associated with vulnerability across LEPH organizations, although it must be acknowledged that mental health problems are not to be viewed as synonymous with vulnerability, as detailed in the Adult Support and Protection Act (2007) (The Scottish Government, 2018). The years 2010 and 2013 were also of particular relevance to the current review from a healthcare and emergency services policy perspective.

The year 2010 was selected because from a Public Health perspective, the Healthcare Quality Strategy for NHS Scotland was published then. This strategy promotes partnership working between key NHS stakeholders including service users (patients, carers, general public) and service providers (local authorities, third sector and the NHS). It aims to provide excellent health services to service users in Scotland (The Scottish Government, 2010). Similarly, the Police and Fire Reform (Scotland) 2012 Act was operational in 2013 and involved merging of policing, and fire and rescue services (The Scottish Parliament, 2012). The reform aims to ensure increased equity of access to specialised services while protecting and enhancing service delivery; improve national capacity in times of crises (for example flooding); and strengthening relationships between service users and providers by promoting the engagement of local councillors in designing and integrating local services with communities (The Scottish Government, 2017). Furthermore, the purpose of policing enshrined within the Police and Fire Reform Scotland Act (2012) is to improve safety and wellbeing by working in collaboration with others, further emphasising the need for shared understandings across working partners in LEPH.

In line with the research question and sub-questions, the articles selected were limited to those which discussed vulnerability including its assessment. This was considered within the context of LEPH. Vulnerability is perceived differently in children and adults within legal definitions. Therefore, focusing on a specific demographic, namely the adult population prevented ambiguity in the research results. While in Scotland, the legislation considers vulnerable adults to be those aged 16 years and over (Adult Support and Protection Act, 2007), this is not the commonly held stance on adulthood internationally, with the majority of countries considering adulthood as 18 years and older. We therefore decided to adopt the wider-adopted 18 years and older definition within the current scoping review to allow international consistency across the literature searching and inclusion. CINAHL, MEDLINE, PsycINFO, Criminology Collection, and Sociology Collection were selected as key databases because they contain articles that address LEPH matters.

#### Exclusion criteria

This research is targeted at a global audience including LEPH departments, governments/policy makers, and academic researchers. Accordingly, the research findings are intended to:

- Raise global awareness of issues relating to vulnerability identification and assessment across LEPH departments. Since vulnerability assessment is a growing concern across LEPH departments, we believe that it is more expedient to focus on findings from contemporary studies which might reflect this new reality; hence the exclusion of papers published before the year 2000;
- Guide Governments in strategic decision-making. As government policies and strategic plans typically span a 10-year period, it is necessary to consider contemporary studies focusing on vulnerability definitions and assessments. This is another reason why we excluded papers published prior to 2000.

To some extent, some of the other exclusion criteria (articulated next) constitute research limitations. First, articles published in a language other than English were excluded. This was due to funding and time limitations, including the lack of a multi-lingual member in our six member research team. In so doing, we acknowledge that some relevant papers may have been excluded.

Second, the adult age as articulated in the exclusion criteria, is from 18 years and above. As the Adult Support and Protection Legislation in Scotland categorises people from 16 years and above as adults (Care Information Scotland, 2018), the findings of this review may exclude young adults between 16 years old and those just under 18 years old; constituting a limitation. However, as detailed earlier, the need to consider the international context, rather than only the local context of the authors, was considered desirable for the current review, and as the majority of international legislation considers adulthood to begin at 18 years old, we chose this upper threshold. That said, we would encourage future authors to consider carefully whether to expand the definition of adulthood to begin at 16 years old. Similarly, grey literature was not included because these are not usually peer reviewed.

Third, articles that did not explicitly mention the word ‘vulnerability’ in their abstract were excluded. As our key focus was on vulnerability, we felt that articles that did not mention vulnerability specifically in their abstract might not discuss vulnerability as thoroughly as required to address out research question. The key purpose of the paper was to identify definitions of vulnerability across LEPH; the use of synonyms to depict vulnerability was considered as a potential confounding factor. Therefore, including only papers with vulnerability in the abstract and which later discussed vulnerability as a construct in depth allowed for definitions across LEPH to be drawn out and considered. For this reason, papers that failed to discuss vulnerability form a LEPH perspective in the body of the article were also excluded. We agree that some relevant papers may have been excluded due to the vagueness of the term in everyday language use, and restrictions to abstract length and content in some journals. Thus, to some degree, the abstract screening constitutes a limitation. We also acknowledge the relevance of bringing together a unified ‘language’ for understanding vulnerability as a concept. Still, trying to encapsulate every potential descriptor for vulnerable people would be outside the scope of the current scoping review and could be a piece of work in its own right.

Fourth, although there are some excellent peer reviewed book chapters published, many are also not peer reviewed. Peer reviewed articles are typically reviewed by academics, contain subject-relevant terms, subjected to a thorough assessment process and are targeted at researchers and professionals. Book chapters and non-peer reviewed articles were excluded because they do not always meet these criteria. Due to the heterogeneity of peer review and the absence of a process to identify peer reviewed book chapters, we chose to omit all book chapters from this scoping review. Nonetheless, the decision to include only known peer reviewed sources may have led to unintentional exclusion of some relevant sources.

Considering these limitations, we suggest that subsequent reviews should consider including: publications in languages other than English, grey literature to enable deeper insight into vulnerability assessments from LEPH perspectives; synonyms of vulnerability during the search for relevant articles; and book chapters.

### Stage 3: Selecting the studies

In applying the inclusion and exclusion criteria, boundaries were established which aided in the selection of relevant studies. See Appendix for the CINAHL, MEDLINE, PsycINFO search criteria which we conducted via the EBSCO platform. As limits were not placed on the country of study or publication, studies from different countries were included in the review. Thus, vulnerability definitions and assessments could be identified from different geographical contexts, enabling analytical breadth and international relevance.

### Stage 4: Conducting content analysis

Relevant articles were exported from CINAHL, MEDLINE, PsycINFO, Criminology Collection, and Sociology Collection into *Endnote* reference management software for storage and referral purposes. Following title and abstract screening, the remaining papers were subsequently exported to *NVivo* (qualitative data analysis software), to enable effective, efficient and transparent content analysis. Specifically, a *Text Search Query* was conducted to retrieve discussions on vulnerability. The findings included the following headings:

- Author name and date
- Article title
- Journal name
- Research country
- Research context (Law Enforcement, Public Health, or both)
- Discussions involving definitions of vulnerability and brief descriptions of vulnerability assessment, if any
- Vulnerability associations
- Research gaps.

### Stage 5: Recording, organising and summarising the result

#### Recording the result

As indicated in Fig. 1, 155 records were identified by searching through the five key databases. Eight duplicates were removed. Following the application of the inclusion and exclusion criteria, an additional 113 records were removed. Of these 113 records, 30^4^ were removed because they were inaccessible, eight because their titles did not align with the research question, and 73 because their abstracts did not refer to ‘vulnerable’ or ‘vulnerability’. After the full paper reading of the remaining papers, two were removed because they failed to address the research question. The 34 remaining records met the inclusion criteria and were considered eligible for screening and content analysis via *NVivo 10*. The following sections organize the results in terms of vulnerability definitions and vulnerability assessment.

#### Organising the Results

##### Vulnerability definitions

The scoping review revealed that definitions of vulnerability are at best fragmented, with only four of the 34 reviewed articles providing explicit definitions of vulnerability as indicated in Table 2.

**Table 2:** Explicit Vulnerability (Vuln) Definitions (4). Presentation of articles with explicit definitions of vulnerability

#	Author/Name/Date	Research Country	Research Context	Vuln Definitions	Vuln Associated with
1	Damsere-Derry et al. (2017)	Ghana	Law Enforcement (criminal justice) and Public Health	Vulnerable road users (VRU) are defined as those who are exposed to the risk of traffic accidents because they lack protective frames. They include pedestrians (over-speeding), cyclists and motorcyclists (failure to use helmet). Of the three groups, pedestrians have higher risks of injuries or deaths owing to an absence of any protective frame. Cyclists and motor cyclists are protected to some extent by their protective clothing and helmets. VRU differ from protected road users because they are sheltered by their vehicles and associated devices like airbags, child restraints and seat belts.	Risk of death
2	McNeil & Small (2014)	No specific country - Systematic Literature Review and Meta-Analysis.	LE (safer environments interventions); PH	Vulnerable groups defined and identified as injection drug users. Vulnerability seemed to be used as a synonym for susceptible For example, "vulnerability to health harms..." (p.151), and "to drug-related harms" (p.152).	Social and physical risk environments including contextual factors like social, environmental and structural factors.
3	Whitelock (2009)	UK	PH (mental health)	A 'vulnerable adult' is defined as a person ‘who is or may be in need of community care services by reason of mental or other disability, age or illness; and who is or may be unable to take care of himself, or unable to protect him or herself against significant harm or exploitation’ (Department of Health, 2000, pgs. 8 & 9). Vulnerable adult identified as those with mental health challenges at risk of abuse within mental health wards and their communities	Feeling at risk of abuse, level of risk, experience of abuse, breakdown,
4	Wilson (2016)	Australia	LE (criminal justice - police, court services); PH (social care)	Vulnerability defined based on age, adaptive behaviour, IQ, inappropriate agreement to irrational requests (Nettlebeck & Wilson, 2002).	Weakness and helplessness

##### Vulnerability assessment

The scoping review showed that models for assessing vulnerability lack uniformity across LEPH because it is prioritised differently across these organizations. Tables 3, 4 and 5 show this in more detail. From a Law Enforcement perspective, only one model for vulnerability assessment was identified (See Table 3). It was based on how likely individuals think they may be suitable crime targets, and their ease of accessing social support (Gaitan & Shen, 2018). The assessment model indicated that vulnerability was associated with poverty and perceptions of risk. From a Public Health perspective, five different models for vulnerability assessment were identified. These include:

- Psychosocial Recovery and Development in East Timor (PRADET) (Amiral et al. (2004)
- Rhodes et al.’s (2005, 2012) framework of socio-structural vulnerability
- The use of self-reporting (Thorpe et al., 2011)
- The use of Critical Incident Inventory (CCI) which measures exposure to critical incidents (Ward et al., 2006)
- The use of vulnerability definitions (Whitelock, 2009)

**Table 3:** Models for Assessing Vulnerability (Vuln) - Law Enforcement Perspective. Presentation of articles containing models for assessing vulnerability from a Law Enforcement perspective

#	Author/Name/Date	Research Country	Research Context	Model(s) for Vuln Assessment	Vuln Associated with
1	Gaitan & Shen (2018)	Mexico	LE (Criminal Justice)	Vulnerability assessed based on general indicators like the individual’s perceptions as suitable crime targets, inadequate social support, and incivilities.	Poverty. Vulnerability associated with reduced sense of well-being manifested as poverty. Feelings of vulnerability reduced/dissipated by the perception of increased social cohesion and accessible social resources. Recursive and interconnected relationship between behaviour on one hand and vulnerability and risk perceptions on the other hand.

**Table 4:** Models for Assessing Vulnerability (Vuln) - Public Health Perspective. Presentation of articles containing models for assessing vulnerability from a Public Health perspective

#	Author/Name/Date	Research Country	Research Context	Model(s) for Vuln Assessment	Vuln Associated with
1	Amiral et al. (2004)	East Timore (Post-emergency phase within post-conflict and post-war, low-income developing countries)	PH - Mental Health	The PRADET (Psychosocial recovery and Development in East Timor) was established to assess social vulnerabilities in mental health patients within post-emergency, post-conflict and post-war East Timore.	Mental disturbances and social risk
2	Knight et al. (2014)	US - San Francisco	PH - Mental Health	Rhodes et al.'s (2005, 2012) framework of socio-structural vulnerability (p.8). The authors have adapted the framework to assesses how single room occupancy hotels affect the mental health of women in their capacity as "mental health risk environments"	Risk environment
3	Thorpe et al. (2011)	US - Wisconsin	PH- Healthcare Access	Use of self-report to assess vulnerable elderly people with mental health issues or functional disabilities. Self-report was administered by doctors who asked questions that enabled them identify conditions related to mental health issues. The Health Utilities Index Mark III is used to assess functional health disabilities. Dichotomous variables signifying the existence of reported limitations regarding cognition, dexterity, hearing, speech, ambulation and pain were created by the authors.	Access to healthcare
4	Ward et al. (2006)	South Africa	PH - Prehospital emergency and associated mental health outcomes	Exposure to critical incidents assessed by the authors using f, which is a 22-point scale. The Revised Impact of Event Scale was used after the CII to assess post-traumatic stress disorder (PTSD).	Critical incident disorder and PTSD
5	Whitelock (2009)	UK	PH - Mental Health	Vulnerability assess based on the vulnerability definition provided in the No Secrets Guidance of the Department of Health which considers people's identity, diagnosis, personal characteristics or service eligibility.	Feeling at risk of abuse, level of risk, experience of abuse, breakdown,

**Table 5:** Models for Assessing Vulnerability (Vuln) - LEPH. Presentation of articles containing models for assessing vulnerability from Law Enforcement and Public Health perspectives

#	Author/Name/Date	Research Country	Research Context	Model(s) for Vuln Assessment	Vuln Associated with
1	Beach et al. (2013)	US - New York	LE (arrest, incarceration, forensic, community treatment); PH (psychiatric hospitalization)	Four risk factors used to assess and predict vulnerability. They include the risk of incarceration or arrest, homelessness, premature discharge from assertive community treatment, psychiatric hospitalization.	Forensic histories and high-risk population.
2	Cohen (2016)	US	LE (counter-terrorism, homeland security); PH (mental health)	The prevention approach which is currently adopted by some local communities. It assesses vulnerable individuals by identifying those at risk of committing acts of terrorism and intervenes before it happens. Law enforcement encouraged to work with mental health at this early stage. (Risk management and collaborative working - co-creation of value)	The paper recommends that investigative and violence prevention protocols by federal agents should include behavioural risk assessment techniques. Also multidisciplinary teams comprising community, law enforcement, and mental health should be established to encourage holistic, structured and cohesive collaboration.
3	Damsere-Derry et al. (2017)	Ghana	LE (police); PH	Vulnerability assessed based on level of exposure to the risk of traffic-related injuries and death.	Risk of death
4	Kerr & Jackson (2016)	US	LE (Criminal Justice - inequitable sentencing and policing); PH (Impact of drug war on HIV vulnerability)	Based on three pathways, the authors developed a Drug War AIDS/HIV inequities model developed to assess how HIV vulnerability in African Americans can increase as a result of the drug war. The pathways are sexual networking, social marginalization and resource deprivation.	HIV
5	Slade et al. (2016)	UK - England and Wales	LE (Criminal Justice); PH (mental health)	Mental health vulnerability assessed via the National improvements in prison mental health services, and diversion and liaison services provided in police stations and courts. Based on clinical need, liaison services include communications with hospitals, communities, or prison services. Diversion services involves referring identified vulnerable groups to either a hospital bed or community service.	Mental health disturbances
6	Wilson (2016)	Australia	LE (criminal justice - police, court services); PH (social care)	Appropriate screening, Vulnerability was also assessed using the Social Vulnerability Questionnaire, developed by Fisher, Moskowitz, & Hodapp, 2012; the Test of Interpersonal Competence and Personal Vulnerability, developed by Wilson, Seaman, & Nettelbeck, 1996; and the Decision-making Video Scale, developed by Hickson, Khemka, Golden, & Chatzistyli, 2008.	Weakness and helplessness

Within this context, vulnerability was associated with mental health, social risk, risk environment, risk of abuse, level of risk, access to health care, experience of abuse, and breakdown. From a LEPH perspective, six different models for vulnerability assessment were identified. They include:

- The use of risk factors like:
  - The risk of incarceration or arrest (Beach et al., 2013; Saddichha et al., 2014)
  - The risk of homelessness (Beach et al., 2013; Glynn et al., 2014)
  - The risk of premature discharge from assertive community treatment (Beach et al., 2013)
  - The risk of psychiatric hospitalization (Beach et al., 2013)
- The use of risk factors to identify those at risk of committing acts of terrorism (Cohen, 2016)
- Level of exposure to the risk of traffic-related injuries and death (Damsere-Derry et al., 2017)
- Drug War AIDS/HIV inequities model (Kerr & Jackson, 2016)
- National improvement reports in prison mental services provided in police stations and courts (Slade et al., 2016)
- Appropriate screening although the type of screening was not specified (Wilson, 2016)

According to our findings, the countries with models for assessing vulnerability were Australia, Canada, East Timor, Ghana, Northern and Southern America (Mexico), South Africa and the UK. This is captured in Tables 3, 4 and 5.

Despite the varying models of assessment across LEPH, the use of risk factors to assess vulnerability appeared in three of the six models identified, as captured in Table 5. From this perspective, vulnerability was associated with forensic histories and high-risk population, risk of death, HIV, mental health, feelings of weakness and helplessness.

#### Summarising and discussing the results

As illustrated in Table 6, the current review reveals conflicting priorities across LEPH in relation to vulnerability. Essentially, vulnerability is context-specific from a Law Enforcement perspective, and person-specific from a Public Health perspective.

**Table 6:** LEPH Conflicting Priorities on Vulnerability (Vuln) Issues. Presentation of articles evidencing conflicting priorities across LEPH as regards vulnerability

#	Law Enforcement	References	Public Health	References
1	Criminal Justice	Frye and Dawe (2008); Saddichha et al. (2014); Pinedo et al. (2017); Gaitan & Shen (2018); Hyatt & Han (2018)	Physical Health (HIV)	Simic & Rhodes (2009); Syvertsen et al. (2014); Forbes (2015)
2	Hostage Taking	Ludwig-Barron et al. (2015)	Mental Health	Amiral et al. (2004); Ward et al. (2006); Whitelock (2009); Knight et al. (2014)
3	Intimate Partner Violence	Ludwig-Barron et al. (2015)	Paramedics/ Pre-hospital Emergency	Ward et al. (2006)
4	Racial Profiling and Traffic Stop Risk	Miller (2009)	Healthcare Access	Thorpe et al. (2011)

Studies and discussions on vulnerability from a Law Enforcement perspective revolved around contextual issues. These related to criminal justice, hostage taking, intimate partner violence, racial profiling and traffic stop risk. These issues relate to a specific circumstance, situation and/or place (Table 6). On the other hand, vulnerability studies and discussions from a Public Health perspective addressed personal matters. These relate to patients’ physical health, mental health and access to pre-hospital emergency services and/or health care (Table 6).

At the intersect of LEPH, the selected studies looked at a range of criminal justice and public health issues in tandem. As captured in Table 7, these include but are not limited to policing practices police contact/custody, inequitable sentencing, arrest, incarceration/correctional setting, community treatment, psychiatric hospitalization, parole, forensic, counter-terrorism, victimisation, public health systems, learning disabilities, drug users, court cases, social care and others.

**Table 7:** Vulnerability-related issues at the Intersect of LEPH. Presentation of articles discussing vulnerability-related issues at the intersct of LEPH organisations

#	Law Enforcement & Public Health	References
1	Arrest, incarceration, forensic, community treatment, psychiatric hospitalization	Beach et al. (2013)
2	Policy and public health	Boeri et al (2009)
3	Criminal justice and paramedics	Borschmann et al. (2017)
4	Counter-terrorism, homeland security, mental health	Cohen (2016)
5	Traffic injury prevention	Damsere-Derry et al. (2017)
6	Criminal justice and mental health	Ferrazzi & Krupa (2016)
7	Correctional setting and physical health (HIV)	Frisman et al (2008)
8	Correctional setting and mental health (dementia and cognitive impairment)	Gaston (2018)
9	Criminal justice, public health systems and clinicians	Glynn et al (2014)
10	Parole, probation and drug addictive behaviours	Hall et al (2016)
11	Inequitable sentencing, impact of drug war on HIV vulnerability	Kerr and Jackson (2016)
12	Safer environments and drug users	McNeil & Small (2014)
13	Crime and mental health	Morrall et al (2010)
14	Policing practices and drug injecting deported migrants	Pinedo et al. (2017)
15	Incarceration, substance abuse and mental health	Saddichha et al. (2014)
16	Violence, abuse, learning disabilities	Olszowski & Boaden et al (2010)
18	Incarceration and mental health	Slade et al. (2016)
19	Incarcerated rural women, mental health, HIV	Staton-Tindall et al (2015)
20	Police contact, police custody, mental health (cognitive disability)	Trofimovs & Dowse (2014)
21	Victimisation, court services and social care	Wilson (2016)
22	Learning Disability and risk of criminality	Allen (2007)

Essentially, the studies demonstrated that the concept of vulnerability from a LEPH perspective was wide; extending well beyond the concept of mental health. This probably explains the inconsistencies and lack of explicitness in vulnerability definitions and assessments across LEPH. Likewise, the studies captured in Table 7 confirm that partnership working between policing and public health is unavoidable and necessary.

#### Stage 6: Stakeholder engagement

Stakeholder engagement in this project was in the form of an Expert Advisory Group (EAG). This collaborative partnership comprises 26 individuals within senior roles across LEPH organisations in Scotland, including: Directors, Co-Directors, and Professors of Law Enforcement and Public Health; senior Officers in Police Scotland; senior Public Health Officials across psychiatry, emergency medicine, and substance misuse; Senior members in the Scottish Government; Senior members of voluntary sector organisations and those with lived experience; and academics and researchers working across criminal justice, psychology, health, and vulnerability. The primary purpose of the EAG is to “to inform and support the development of a co-constructed programme of research crossing the intersect of Law Enforcement and Public Health” (Murray et al., 2018, p.1). A follow up EAG vulnerability sub-committee meeting was held to specifically inform the search strategy and research question for the current review, and to identify possible future steps and areas for research which would be a priority in LEPH practice.

## Concluding remarks

Considering LEPH organisations, this review aimed to identify the ways in which vulnerability is defined and assessed across adult populations. The implications of the findings of the current scoping review are two-fold. For “vulnerable groups”, the lack of an evidence-based definition and assessment could introduce a raft of problems. These include preventing access to relevant LEPH services; exacerbating issues of multiple vulnerabilities, co-morbidity, and/or dual diagnosis; and impeding effective communication across LEPH partners. All could inadvertently enable the social exclusion of vulnerable groups from political discourse and policy interventions. For LEPH organizations and, by extension, Federal Governments, the inconsistencies in vulnerability definitions and assessments may result in reactive crisis responses as opposed to proactive preventative measures.

During the scoping review, research gaps were identified. From a co-production and social innovation perspective, Whitelock (2009) identified the absence of a personalised definition of vulnerability. The author stressed the need to develop one that includes the service user’s voice as a critical step towards the care planning and support process. Similarly, Forbes (2015) argued for the need to explicitly identify marginalised sex-workers as vulnerable people. This may increase their chances of being included in political health discourse and could facilitate the development of effective care pathways.

Considering mental health issues, Borschmann et al. (2017) noted the need for further research on clinical management and epidemiology of reactions to self-harm, clinical outcomes and care pathways for vulnerable patients. Likewise, Cohen (36) noted the absence of behavioural risk assessment techniques and recommended that terrorism violence prevention protocols should include such. Recommendations also included the need for more multidisciplinary teams across community, policing and mental health to encourage holistic and structured collaboration and co-production of services (Cohen, 2016).

From an academic perspective, the scoping study clearly exposes the complexities associated with defining and assessing vulnerability across LEPH. This may be because they are perceived and prioritised differently in both organizations. Future research should attempt to bridge this gap. This could assume the form of a Systematic Literature Review to identify *effective* models that are currently used to assess vulnerability in LEPH practice. This would be useful in both academia and in practice. The proposed Systematic Literature Review should form the basis of any future intervention or vulnerability/risk assessment development work to ensure rigour and sound operational and theoretical underpinnings. A synthesis of vulnerability models would enable the development of a vulnerability and mental health assessment framework, for example, which could then be tested across LEPH practice.

The Scoping Review also highlights the challenges associated with *implementing* a universal definition of vulnerability across LEPH organisations. Considering that the goal is to find some commonality with “vulnerable” groups along with policy (Police Scotland, 2017; Van Dijk & Crofts, 2017; Punch & James, 2016), this proposed universal definition would have to be agreed upon by both law enforcement and public health areas since they are two separate entities. We believe that a universal definition would be helpful for a range of law enforcement and public health services and treatment, including the police, courts, control rooms and emergency healthcare. From a LEPH perspective, a universal definition of vulnerability can facilitate universal vulnerability assessment, decision-making processes, and understanding of problems faced across LEPH. Basically, with a shared language in the first instance, and shared understanding of each organisation’s role in the ‘system’, shared decision-making protocols and processes, can be developed. This increases the likelihood of successful and effective partnership working across LEPH.

Ultimately, the ambition is the development of properly linked services, which respect and understand each organisation’s role, strength and limitation, and which takes cognizance of mental health and vulnerability issues. Of course, a whole-systems approach to LEPH is ambitious, but we feel that taking it step by step, starting with shared understanding and definitions is a good step forward, together, across the intersect of LEPH. Also, existing assessment models may need to be reviewed and revised to capture the new, more universal definition of vulnerability if or when it is developed.

## Appendix

**Table 8:** CINAHL/MEDLINE/PsychINFO Search strategy - August 2018

**#**	Query	Results (N Papers)
1	*AB law enforcement OR AB police OR AB policing OR AB criminal justice*	57,862
2	*AB public health OR AB mental health OR AB disparity*	650,689
3	*AB social determinants of health OR AB socioeconomic factors OR AB social inequality OR AB inequality*	72,184
4	*AB adult protective services OR AB adult protection OR AB incapacity OR AB learning disability OR AB learning disorder*	38,771
5	*S2 OR S3 OR S4*	743,471
6	*AB vulnerab* OR AB access*	583,379
7	*AB risk* OR AB at risk*	2,526,889
8	*S1 AND S5 AND S6 AND S7*	29

## Footnotes

1 To understand how and why a term is used, by whom and in what literature.

2 To carve out relevant literature according to scope. Usually involves synthesizing findings from various studies.

3 To identify key documents from the public and private sector that concern practice in the related sector.

4 The cases were excluded after efforts to retrieve them using three Scottish University Library subscriptions (Edinburgh Napier University, Glasgow Caledonian University, University of Edinburgh), google scholar and google search engine failed to provide access to these papers. The decision to include papers that could be reasonably accessed was contingent on the fact that LEPH professionals would likely have even less institutional access to peer reviewed papers. We therefore considered these 30 papers inaccessible to most academics and those working in LEPH practice.
